# Prediction of Neurodevelopment in Infants With Tuberous Sclerosis Complex Using Early EEG Characteristics

**DOI:** 10.3389/fneur.2020.582891

**Published:** 2020-10-16

**Authors:** Jessie De Ridder, Mario Lavanga, Birgit Verhelle, Jan Vervisch, Katrien Lemmens, Katarzyna Kotulska, Romina Moavero, Paolo Curatolo, Bernhard Weschke, Kate Riney, Martha Feucht, Pavel Krsek, Rima Nabbout, Anna C. Jansen, Konrad Wojdan, Dorota Domanska-Pakieła, Magdalena Kaczorowska-Frontczak, Christoph Hertzberg, Cyrille H. Ferrier, Sharon Samueli, Barbora Benova, Eleonora Aronica, David J. Kwiatkowski, Floor E. Jansen, Sergiusz Jóźwiak, Sabine Van Huffel, Lieven Lagae

**Affiliations:** ^1^Pediatric Neurology, Department of Development and Regeneration, University of Leuven KU Leuven, Leuven, Belgium; ^2^Department of Electrical Engineering (ESAT), STADIUS Centre for Dynamical Systems, Signal Processing and Data Analytics, KU Leuven, Leuven, Belgium; ^3^Department of Neurology and Epileptology, The Children's Memorial Health Institute, Warsaw, Poland; ^4^Child Neurology and Psychiatry Unit, Systems Medicine Department, Tor Vergata University, Rome, Italy; ^5^Child Neurology Unit, Neuroscience and Neurorehabilitation Department, “Bambino Gesù” Children's Hospital, Rome, Italy; ^6^Department of Child Neurology, Charité University Medicine Berlin, Berlin, Germany; ^7^Neuroscience Unit, Queensland Children's Hospital, Brisbane, QLD, Australia; ^8^University of Queensland School of Clinical Medicine, Brisbane, QLD, Australia; ^9^Department of Pediatrics, Medical University Vienna, Vienna, Austria; ^10^Department of Paediatric Neurology, Charles University, Second Faculty of Medicine, Motol University Hospital, Prague, Czechia; ^11^Department of Pediatric Neurology, Reference Centre for Rare Epilepsies, Imagine Institute, Necker- Enfants Malades Hospital, University Paris Descartes, Paris, France; ^12^Pediatric Neurology Unit, Universitair Ziekenhuis Brussel, Brussels, Belgium; ^13^Transition Technologies, Warsaw, Poland; ^14^Institute of Heat Engineering, Warsaw University and Technology, Warsaw, Poland; ^15^Diagnose und Behandlungszentrum für Kinder und Jugendliche, Vivantes Klinikum Neuköln, Berlin, Germany; ^16^Department of Child Neurology, Brain Centre, University Medical Centre Utrecht, Utrecht, Netherlands; ^17^Department of (Neuro)Pathology, Amsterdam Universitair Medisch Centrum, University of Amsterdam, Amsterdam, Netherlands; ^18^Stichting Epilepsie Instellingen Nederland (SEIN), Heemstede, Netherlands; ^19^Harvard Medical School, Brigham and Women's Hospital, Boston, MA, United States; ^20^Department of Child Neurology, Medical University of Warsaw, Warsaw, Poland

**Keywords:** tuberous sclerosis complex (TSC), EEG, biomarker, neurodeveloment, autism (ASD), TAND profile

## Abstract

Tuberous Sclerosis Complex (TSC) is a multisystem genetic disorder with a high risk of early-onset epilepsy and a high prevalence of neurodevelopmental comorbidities, including intellectual disability and autism spectrum disorder (ASD). Therefore, TSC is an interesting disease model to investigate early biomarkers of neurodevelopmental comorbidities when interventions are favourable. We investigated whether early EEG characteristics can be used to predict neurodevelopment in infants with TSC. The first recorded EEG of 64 infants with TSC, enrolled in the international prospective EPISTOP trial (recorded at a median gestational age 42 4/7 weeks) was first visually assessed. EEG characteristics were correlated with ASD risk based on the ADOS-2 score, and cognitive, language, and motor developmental quotients (Bayley Scales of Infant and Toddler Development III) at the age of 24 months. Quantitative EEG analysis was used to validate the relationship between EEG background abnormalities and ASD risk. An abnormal first EEG (OR = 4.1, *p*-value = 0.027) and more specifically a dysmature EEG background (OR = 4.6, *p*-value = 0.017) was associated with a higher probability of ASD traits at the age of 24 months. This association between an early abnormal EEG and ASD risk remained significant in a multivariable model, adjusting for mutation and treatment (adjusted OR = 4.2, *p*-value = 0.029). A dysmature EEG background was also associated with lower cognitive (*p*-value = 0.029), language (*p*-value = 0.001), and motor (*p*-value = 0.017) developmental quotients at the age of 24 months. Our findings suggest that early EEG characteristics in newborns and infants with TSC can be used to predict neurodevelopmental comorbidities.

## Introduction

Tuberous sclerosis complex (TSC) is an autosomal dominant disorder affecting ~1 in 5,800 individuals (1). This disorder is caused by loss of function mutations in the tumour-suppressor genes *TSC1* or *TSC2*, encoding the proteins hamartin and tuberin. Both proteins are key components of a TSC protein complex that regulates the state of activation of the Ras homolog enriched in brain GTPase and hence the mammalian target of rapamycin complex 1 (mTORC1) (2, 3). Overactivation of mTORC1 results in disorganized cellular growth, differentiation, metabolism, and impaired autophagy, leading to the formation of hamartomatous lesions in various organs, causing a multisystem disorder (2). However, the clinical features of TSC are both age-dependent and highly variable (2).

In patients with TSC, both epilepsy and neurodevelopmental comorbidities including intellectual disability and autism spectrum disorder (ASD) are common. Epilepsy affects 80–90%, 6% of TSC infants develop seizures during the first month of life, and 60–70% develop epilepsy within the first year of life (4–7). About 60% of TSC patients develop drug-resistant epilepsy (4, 8). In addition, 40–50% of TSC patients have intellectual disability. ASD is diagnosed in 21–50% of patients with TSC (9–12). In TSC several characteristics are associated with intellectual disability and ASD, including having a *TSC2* mutation, young age at seizure onset, a high seizure burden, epileptic spasms, and drug-resistant epilepsy (6, 13–16). Similarly, TSC patients with intellectual disability have a higher prevalence of epilepsy (88%) and a higher rate of drug-resistant epilepsy (65%) (1, 2).

Recent studies have shown that early interventions can improve seizure control and can preserve neurodevelopment to some extent (13, 17). A small non-randomized pilot clinical trial of early intervention with vigabatrin at the time of multifocal interictal epileptiform discharges (IED) detection, led to a significant reduction in drug-resistant epilepsy, more seizure-free patients, and fewer patients with intellectual disability at 24 months in comparison to an historical control group in which vigabatrin was started only after seizure onset (18). More recent follow-up of these TSC children showed persistent improvement at school age (19).

Prenatal or early postnatal diagnosis of TSC is currently possible, due to the visualisation of cardiac TSC related-lesions or cortical tubers (2). Since early diagnosis is feasible, TSC is an interesting disease model to study both epileptogenesis and investigate potential biomarkers of neurodevelopmental comorbidities. Early abnormal EEG activity had been shown to be predictive for later epilepsy. Abnormal EEG activity can be observed before the development of clinical seizures in TSC (20). In a prospective study of 32 infants with TSC, 20 patients developed seizures. In 17/20 IED preceded the onset of seizures, by an average interval of 3·6 months (21) A recent quantitative EEG study showed that increased EEG connectivity in infants with TSC preceded the onset of epileptic spasms (22).

In this exploratory EEG study we investigated whether the first EEG, recorded in infants with TSC, and taken below the age of 4 months could be used to predict neurodevelopment and especially ASD risk at the age of 24 months. Our hypothesis was that early EEG characteristics, such as IED and background abnormalities can predict neurodevelopmental outcome. In addition to the visual analysis of the EEG, we used quantitative EEG methodology for assessment and quantification of EEG background abnormalities.

## Patients and Methods

### Patients

This EEG study was part of the EPISTOP project. The EPISTOP project was a multicentre long-term, prospective study evaluating clinical and molecular biomarkers of epileptogenesis in TSC (NCT02098759). As part of this biomarker study, serial EEGs were collected during this project. A second aim of the EPISTOP study was to investigate the potential benefit of preventive treatment with vigabatrin after the appearance of IED on the EEG (focal IED for more than 10% of the recording time, multifocal IED, generalized IED or hypsarrhythmia, assessed by the local electroencephalographer) and before seizure onset vs. conventional follow-up and treatment only after the onset of clinical or electrographic seizures.

Patients were enrolled from November 2013 to August 2016 at 10 sites. Male or female infants of age ≤4 months with a definite diagnosis of TSC (23), but without previous seizures, or antiepileptic treatment were enrolled after informed consent of their caregivers, which was obtained in accordance with the Declaration of Helsinki. The EPISTOP study was approved by local ethical committees at all study sites.

### EEG

The EEG was recorded for at least 1 h, including wake and sleep at least until stage two. The EEG was performed using a minimum of 19 electrodes according to the 10–20 system. A reduced array with nine electrodes was allowed in infants under 3 months of corrected age (Fp1, Fp2, C3, C4, T4, T3, O1, O2, Cz). The video-EEGs were anonymized, coded and uploaded to a secure central server at the University of Leuven in Belgium. EEG assessment was performed blinded to TSC gene mutation, clinical (with exception of the age of the patient) and outcome data and was done using BrainRT^TM^ software version 3·5 (OSG BVBA, Rumst, Belgium). For this EEG study, the first EEG after enrolment was analysed. All EEGs were analysed separately by three experienced clinical EEG readers. When there was disconcordance, consensus was reached after discussion between the three readers.

For each EEG the presence or absence of IED and electrographic seizures was assessed. An electrographic seizure was defined as an ictal EEG pattern, without clinical correlate and with a duration of at least 10 s (24). Background was scored as follows: normal, focal slowing, and/or a dysmature EEG background. Focal slow wave activity indicates focal cerebral pathology and dysfunction of the underlying brain region (25). A dysmature EEG background was defined as a background inappropriate for the age of the child. Defining characteristics of a dysmature EEG background are: an abnormal discontinuity, persistence of extremely slow delta waves (<2 Hz at term age), asynchrony, or several transient waveforms inappropriate for the gestational age (GA), or poor development of sleep stages according to GA (26–28).

These dysmaturity features can also be analysed by a variety of quantitative approaches (26, 29–31). In this study, four sets of features were derived from the EEGs to describe dysmaturity in young infants (see Supplementary Material):

1. The power in the common EEG frequency bands (δ_1_, δ_2_, θ, α* β*) to assess the persistence of slow waves.
2. Quantitative EEG features derived from the range EEG (rEEG) [an estimation of amplitude-integrated EEG (aEEG)]. Specifically, the difference in distance from the rEEG median from the upper or the lower margin, known as rEEG asymmetry, was used to measure the discontinuity of the signal.
3. With maturation, the EEG background evolves from a discontinuous to a more continuous pattern, showing less regularity and more complexity. This complexity of the EEG signal was measured by means of entropy: the higher the complexity, the higher the entropy (29).
4. The regularity of the EEG signal was assessed by means of the Hurst Exponent: the higher the regularity, the higher the Hurst exponent (30).

### Outcome Measures

Neurodevelopmental outcomes were studied at the age of 24 months. ASD risk was based on the Autism Diagnostic Observation Scale 2 (ADOS-2) score (Toddler Module), which is the gold standard for assessing and diagnosing ASD. If the ADOS-2 test could not be performed due to the child being non-verbal, having a non-verbal age equivalent below 12 months, or was not able to walk independently, Diagnostic and Statistical Manual of Mental Disorders-5 (DSM-5) clinical criteria were applied. Non-verbal children with a total score of 0–9, 10–13, and >13 were classified as “no risk,” “mild/moderate risk,” and “high risk,” respectively. For verbal children the cut-off scores for the three risk categories were 0–7, 8–11, and >11. Cognitive, language and motor developmental quotients (DQ) were based on the Bayley Scales of Infant and Toddler Development III (BSID-III) results at the age of 24 months. Tests were performed by neuropsychologists certified through formal reliability training at each centre and sent to a central research team in Rome for analysis and classification.

### Statistical Analysis

Continuous non-EEG characteristics between groups were assessed by Mann-Whitney *U-*test, because of deviation from the normal distribution demonstrated by Q-Q plots and Kolmogorov-Smirnov test. Both univariable and multivariable linear models were used to assess the relation between EEG characteristics and the DQs on the cognitive, language and motor test of the BSID-III at 24 months. Since the DQs on the cognitive and language test were not normally distributed, the DQs were logarithmic and reciprocal transformed, respectively. Associations between discrete variables and ASD risk were analysed using univariable, and multivariable logistic regression analyses. The selection of non-EEG variables in multivariable models was based on literature search on predicting variables of neurodevelopmental outcome in infants with TSC and based on our univariable models including non-EEG variables (Supplementary Tables 1, 2, 4) (14, 18, 19). The most important non-EEG characteristic considered in multivariable analyses were the mutation and the treatment strategy (preventive or conventional treatment). Due to the low sample size, no interaction terms were added to the model. In multivariable logistic regression models all variables were entered in block. Omnibus tests of model coefficients and Nagelkerke R^2^ are reported below each multivariable logistic regression model. Multicollinearity was assessed for each multivariable logistic regression model. Tolerance values were >0.1 and VIF values were <10, indicating no collinearity between the variables. A two-sided *p*-value < 0.05 was considered statistically significant. Analyses were performed using the Statistical Package for the Social Sciences (SPSS version 26·0; Armonk, NY: IBM Corp.).

#### Quantitative EEG Analysis

For each set of the quantitative EEG features, the most discriminant features were extracted to classify ASD outcome. A binary classification model was developed with linear discriminant analysis (LDA) using three-fold testing, which means that two thirds of patients were used to develop the model and one third to test its performance. The results were reported in terms of misclassification error [percentage of misdiagnosis E(%)] and area under the receiver operating curve (AUC).

## Results

For this EEG study, 64 first EEGs of EPISTOP patients were available for in dept analysis (Table 1). The median GA and chronological age at first EEG were 42 4/7 weeks (IQR [40 2/7–45 2/7 weeks]) and 25 days (IQR [15.25–50.75 days]), respectively. In eighty-four percent of the patients the first recorded EEG was done before a GA of 48 weeks. In 63/64 patients, complete neurodevelopmental follow up was available. In one patient only the cognitive DQ at 24 months was known as the language and motor DQs and ASD assessment at 24 months were missing.

**Table 1:** Baseline and EEG characteristics of the study cohort and neurodevelopmental outcome at 24 months.

	**Overall cohort (*N* = 64)**
**BASELINE**	
GA at birth	38 1/7 weeks (37–40)
Sex	
Male	35 (55%)
Female	29 (45%)
Mutation	
Pathogenic *TSC1* variant	17 (27%)
Pathogenic *TSC2* variant	46 (72%)
No identified variant	1 (1%)
Preventive treatment	19 (30%)
**EEG CHARACTERISTICS**	
Age at first EEG	
GA (weeks)	42 4/7 (40 2/7–45 2/7)
Chronological age (days)	25 (15.25–50.75)
Abnormal first EEG^*^	37 (58%)
Presence of IED	28 (44%)
Focal IED	7 (11%)
Multifocal IED	21 (33%)
Multifocal IED: 1 hemisphere	2 (3%)
Multifocal IED: 2 hemispheres	19 (30%)
Electrographic seizures	6 (9%)
Background abnormalities	23 (36%)
Dysmature EEG background^**^	14 (22%)
Focal EEG slowing	15 (23%)
**NEURODEVELOPMENTAL OUTCOME**	
ASD symptoms (Data available 63/64)	19 (30%)
DQ cognitive BSID-III (Data available 64/64)	75 (65–90.75)
DQ language BSID-III (Data available 63/64)	68 (59–77)
DQ motor BSID-III (Data available 63/64)	73 (67–85)

*Data are n (%) or median (IQR)*.

* *Abnormal first EEG: IED, background abnormalities, or electrographic seizures*.

** *Dysmature EEG characteristics: abnormal discontinuity for the GA (10/14), persistence of high levels of interhemispheric asynchrony inappropriate for the GA (3/14), and extremely slow delta waves (1/14). GA, gestational age; IED, interictal epileptiform discharges; ASD, autism spectrum disorder; DQ, developmental quotient; BSID-III, Bayley Scales of Infant and Toddler Development III*.

**Figure 1:**
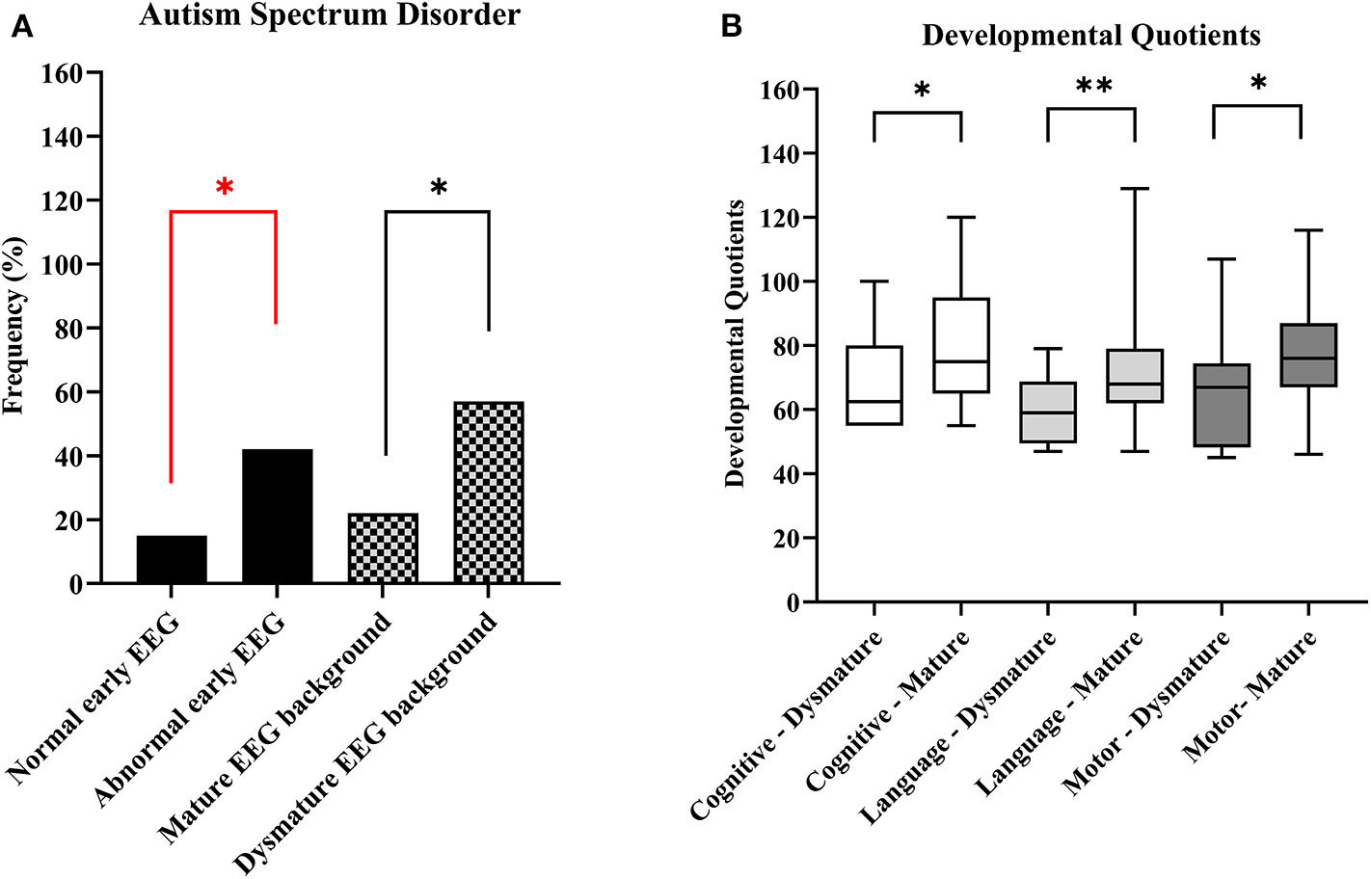
Overview of EEG characteristics and neurodevelopmental outcome at the age of 24 months. **(A)** Autism spectrum disorder by EEG abnormalities. **(B)** Cognitive, language and motor developmental quotients based on Bayley Scales of Infant and Toddler Development -III test results. *p-value < 0.05, **p-value < 0.01. A red line indicates significance in a multivariable model.

Nineteen infants (30%) were diagnosed with ASD traits at the age of 24 months (Table 1). When we assessed the relation between early EEG abnormalities and ASD risk, we found that 15/36 (42%) children with an abnormal first EEG were diagnosed with ASD symptoms at the age of 24 months, compared to 4/27 (14%) of the children with a normal first EEG. In a univariable logistic regression analysis, an abnormal first EEG was significantly associated with a higher probability of ASD at the age of 24 months (*p*-value = 0.027). The odds ratio (OR) was 4.1 (95% CI = [1.2 – 14.4]). In a multivariable logistic regression analysis including the treatment strategy (preventive treatment or conventional follow-up and only treatment after seizure onset) and the pathogenic TSC variant (*TSC1* or *TSC2*) as covariables, an abnormal first EEG remained significantly associated with a higher probability of ASD at the age of 24 months [*p*-value = 0.029, adjusted OR = 4.2 (95% CI = [1.2 − 15.5])] (Table 2, Figure 1). The positive predictive value (PPV), or how often an abnormal first EEG correctly predicted ASD symptoms at the age of 24 months, was 42%. The negative predictive value (NPV), or how often a normal EEG was associated with no ASD symptoms at 24 months of age, was 85% (Sensitivity: 79%, specificity: 52%). Additional analysis of EEG characteristics showed that a dysmature EEG was significantly associated with a higher probability of ASD symptoms at 24 months [8/14 (57%) of patients with a dysmature EEG vs. 22% (11/49) of patients with a mature EEG, univariable *p*-value = 0.017, unadjusted OR = 4.6 (95% CI = [1.3 − 16.1])] (Figure 1). In multivariable logistic regression analysis, the strong association was no longer significant [*p*-value = 0.092, adjusted OR = 6.3 (95% CI = [0.7 − 52.9])] (Table 2). The PPV of a dysmature EEG and ASD traits at the age of 24 months was 57%. The NPV of a mature EEG and no ASD symptoms was 78% (Sensitivity: 42%, specificity: 86%). Other characteristics of the first EEG, such as the presence of IED or focal slowing were not associated with ASD risk at 24 months (Supplementary Table 1).

**Table 2:** Multivariable logistic regression models predicting ASD symptoms at the age of 24 months.

	**B**	**S.E**	***p*-value**	**OR (adjusted)**	**95% CI**
**PREDICTOR VARIABLES OF THE FIRST MULTIVARIABLE MODEL**					
Abnormal EEG	1.446	0.661	0.029	4.2	1.2–15.5
Conventional follow-up and treatment	−0.338	0.655	0.606	0.7	0.2–2.6
Pathogenic *TSC2* variant	−0.210	0.682	0.759	0.8	0.2–3.1
*Overall model χ^2^ (df 3, N = 63) = 5.485 p-value 0.140–R^2^ Nagelkerke = 0.120*					
**PREDICTOR VARIABLES OF THE SECOND MULTIVARIABLE MODEL**					
Abnormal EEG	1.348	0.820	0.100	3.9	0.7–19.2
Abnormal EEG background	−0.842	1.052	0.423	0.4	0.06–3.4
Dysmature EEG background	1.836	1.088	0.092	6.3	0.7–52.9
Conventional follow-up and treatment	−0.758	0.732	0.300	0.5	0.1–2.0
Pathogenic *TSC2* variant	−0.558	0.738	0.449	0.6	0.1–2.4
*Overall model χ^2^ (df 5, N = 63) = 9.014 *p*-value = 0.108–R^2^ Nagelkerke = 0.191*					

*The first multivariable model includes abnormal EEG, conventional follow-up and treatment (infants not receiving preventive treatment) and a pathogenic variant in TSC2 as predictor variables. The second multivariable model includes the EEG background abnormalities and maturation, conventional follow-up and treatment (infants not receiving preventive treatment) and a pathogenic variant in TSC2 as predictor variables. Below the included variables the goodness-of-fit statistic and Nagelkerke R^2^ for the multivariable models are reported in italic. B = regression coefficient, S.E., standard error of the regression coefficient, OR, odds ratio, 95% CI, 95% confidence interval of the odds ratio*.

**Figure 2:**
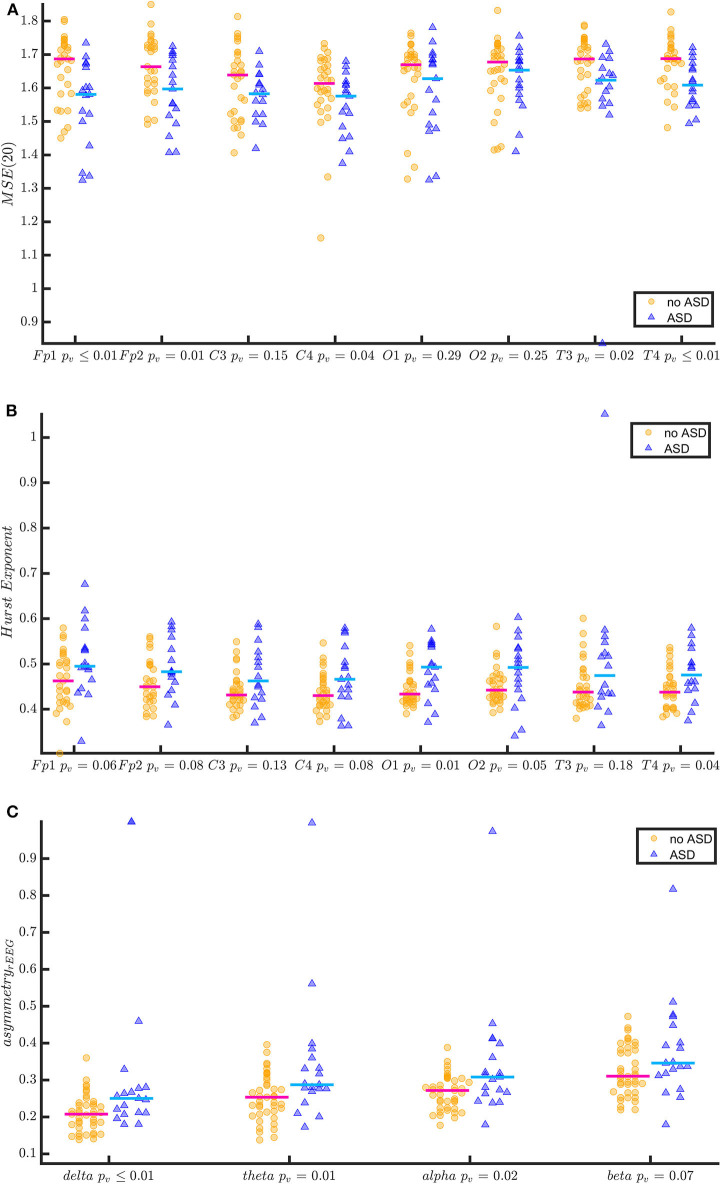
Quantitative EEG features of TSC patients with and without ASD at the age of 24 months. The figure shows the entropy at scale 20 [MSE(20)] **(A)** Hurst Exponent **(B)** and the asymmetry of the range EEG **(C)** (estimate of amplitude integrated EEG) in two groups (no ASD vs. ASD at 24 months). The EEGs of patients with ASD at 24 months show a more asymmetric range EEG, a higher Hurst Exponent (more regularity) and lower entropy at lower frequencies [MSE(20)] (less complexity). In **(A,B)**, the comparisons are reported for each EEG channel. In **(C)**, the comparisons are reported for the frequency bands. *P*-values have been derived by Kruskal-Wallis test.

The quantitative analysis of EEG background confirmed the association of an early abnormal/dysmature EEG, and ASD traits at the age of 24 months. A dysmature EEG is characterised by less complexity, with less entropy and higher regularity. The boxplots in Figure 2 show in TSC patients with ASD traits significantly less entropy [MSE(20)] and higher regularity (Hurst Exponent) compared to children with no ASD risk. Also more asymmetry in the rEEG in the lower frequency bands was found in TSC patients with ASD traits as result of the persistence of slow-waves and discontinuity in the lower frequencies. The discriminatory power of this quantitative analysis to predict ASD traits in infants with TSC was further confirmed by the results of the binary classification models developed with LDA (see Appendix and Supplementary Table 4). The AUC (a measure of classification accuracy) of the different LDA models was in the range of 66–79% (Table 3).

**Table 3:** Results of the binary classification models developed with linear discriminant analysis.

	**E (%)**	**AUC (%)**
Power	32.59	66
Entropy	33.01	79
Asymmetric rEEG	30.26	69
Fractality	22.12	74

*Results of the binary classification models developed with linear discriminant analysis (LDA) for the prediction of ASD symptoms at the age of 24 months. The results are reported in terms of misclassification error [percentage of misdiagnosis, E(%)], and area under the receiving operating curve (measure of classification accuracy, AUC). The different features sets are predictive of ASD symptoms at the age of 24 months with areas under the curve (AUC) close or higher than 70%. rEEG, range EEG*.

The median cognitive, language, and motor DQs based on BSID-III results at 24 months were: 75 (IQR [65 − 90.75]), 68 (IQR [59 − 77]), and 73 (IQR [67 − 85]), respectively (Table 1). Children with an early abnormal EEG background had significantly lower cognitive [median 70 (IQR [55 − 80]) vs. 80 (IQR [67.50 − 95]), *p*-value = 0.029], language [median 59 (IQR [53 − 71]) vs. 71 (IQR [62 − 81.75]), *p*-value = 0.006], and motor (median 70 (IQR [55 − 79]) vs. 76 (IQR [67.50 − 89]), *p*-value = 0.042] DQs at 24 months compared to those with a normal EEG background. The cognitive [median 62·50 (IQR [55 − 80]) vs. 75 (IQR [65 − 95], *p*-value = 0.029], language [median 59 (IQR [49.50 − 68.75]) vs. 68 (IQR [62 − 79]), *p*-value = 0.001], and motor [median 67 (IQR [48.25 − 74.50]) vs. 76 (IQR [67 − 87]), *p*-value = 0.017] DQs were significantly different between the children with a dysmature and those with a mature EEG background at 24 months of age (Figure 1). Using a multivariable linear model, also including other EEG characteristics, as well as the pathogenic TSC variant (*TSC1* or *TSC2*) and the treatment strategy, the results were no longer significant. In these multivariable linear models, a *TSC2* mutation was significantly associated with a lower cognitive and motor DQs at 24 months (Supplementary Tables 3, 5, 6). Other EEG characteristics, such as the presence of IED and focal slowing, were not significantly associated with the cognitive, language, and motor DQs at 24 months of age (Supplementary Tables 2, 4).

## Discussion

The main finding of our study is that an abnormal first EEG in neonates and infants with TSC, and more specifically a dysmature EEG background was associated with a higher probability of ASD traits at the age of 24 months. The sensitivity of this finding was low (42%), but the specificity was high (86%). Hence, an infant with TSC and an EEG in the first weeks of life showing a mature background was less frequently diagnosed with ASD symptoms at 24 months. A dysmature EEG background was also associated with lower DQs on cognitive, language, and motor BSID-III test results. It is important to stress that the predictive value was not influenced by mutation status, or by preventive or standard anti-epileptic treatment during follow up. We were also able to confirm our qualitative findings with a more quantitative approach, which ultimately might become a more reliable biomarker of EEG background development and abnormalities. Our findings are of major clinical importance as an early abnormal EEG might trigger early diagnosis and management of ASD in TSC children.

In TSC, dysregulation of mTOR signalling results in aberrations in cellular morphology, disturbed maturation of the migration patterns of the dysmorphic neurons, altered GABA_A_, and AMPA receptor functions, which contributes to increased seizure susceptibility, epileptic synchronisation, altered synaptogenesis, and altered connectivity. The subsequent malformations of cortical development provide a neuroanatomical and functional substrate for the early appearance of seizures, and developmental, and psychiatric disorders seen in association with TSC (32–35). Neuropathological investigation of brain tissue of foetuses with TSC, stillborn between a GA of 23 to 38 weeks, suggested that mTOR overactivation during embryonic brain development, presumably between 10 and 20 weeks after conception, underlies the formation of brain lesions in patients with TSC (32). In addition, evidence was present for the involvement of the innate and adaptive immune system, which could be responsible for the dynamic changes occurring over time in tubers (32). Besides grey matter pathology in TSC, also the white matter is both on a structural and the neuropathological level affected. White matter radial migration lines have been seen in 20% of patients (9). Moreover, neuropathological studies found white matter pathology with depleted myelin and oligodendroglia in 62% of TSC patients (36). The underlying disturbed architecture and connectivity as a consequence of mTOR overactivation result in a hyperexcitable neural network. The early emergent EEG characteristics of this altered neural network, including dysmaturity, not only reflect the ongoing epileptogenesis in TSC, but can also assist to assess the risk of neurodevelopmental comorbidities.

Benova et al. showed a relation between EEG background abnormalities and ASD, and intellectual disability in a cohort of 22 children with TSC, who were followed from birth until the age of 12 years, although the latter association was not significant (15). Furthermore, in children with TSC aged above 3 years a significant association was found between ASD and the presence of IED, and the number of lobes with IED, but not with focal slowing (11). The abovementioned studies suggest that EEG characteristics, both IED and background abnormalities, are related to neurodevelopment. However, no prospective studies have been performed in neonates and young infants with TSC investigating the early EEG features and their prognostic value for developmental outcome. Neurodevelopmental outcome studies using maturational features of EEG are available in preterm infants. Several studies found that a dysmature EEG background was associated with a poor cognitive outcome (27, 28, 37–39). A recent meta-analysis of 255 young preterm infants born before a GA of 34 weeks and followed with EEGs until a GA of 43 weeks found that a dysmature, or a disorganised EEG pattern predicted cognitive outcome (assessed ≥ 3 months) (40). Although the included studies in the meta-analysis used different definitions, follow-up protocols, and neurodevelopment assessments, these results confirm that the absence of a dysmature or disorganised EEG background is a good predictor of normal cognitive development in preterm infants (40).

The most recent EEG study of Wu et al. enrolled infants with TSC that were older (average age of 3·6 months at enrolment) compared to our patients (average age of 1 month at enrolment) and did not assess the strength of association between EEG features and neurodevelopmental outcome (21). Wu et al. found that IED predicted subsequent epilepsy in 77% of the patients. They also reported that persistent seizures are associated with a decline on the Vineland and Mullen tests at 2 years of age (21). No specific data on ASD risk were reported in this paper (21).

Recent studies using quantitative EEG analysis also facilitate a better understanding of the neurobiological mechanisms of the altered brain maturation in patients with TSC and can help with the identification of infants requiring developmental interventions. Peters et al. found, using graph theory, in older children, adolescents and adults with ASD, with and without TSC, a decreased long- over short-range connectivity with local over-connection and decreased functional specialisation (34). Dickinson et al. showed in patients with TSC, using features of spontaneous alpha oscillations (alpha power, alpha phase coherence, and peak alpha frequency) in high density EEGs, a reduced interhemispheric alpha phase coherence between the left temporal and the right parietal area compared to controls at 12 months, suggesting a delayed or atypical maturation of white matter during infancy (41). In addition, within the group of patients with TSC the reduction in long range interhemispheric alpha phase coherence between the right parietal and left temporal region at the age of 24 months was more pronounced in children with ASD diagnosis (41).

Besides clinical and quantitative EEG features, other characteristics, such as the pathogenic TSC variant, the epilepsy course (including the development of refractory epilepsy or epileptic spasms), MRI features and early development, can help to identify young infants with TSC at risk of developmental comorbidities (6, 12, 16, 42–47).

One of the limitations of our study is that no measure of parental education or parental intelligence was included to predict developmental outcome. Second, the diagnostic power of the neurodevelopmental assessments at 24 months of age is perhaps not optimal, particularly in terms of the stability of diagnostic classification. It is theoretically possible that children who were classified with no ASD risk at 24 months of age are still diagnosed with ASD later in life. A follow-up study of EPISTOP patients with neurodevelopmental assessments at the age of 6 years is planned. Third, our cohort was relatively small, consequently including interaction terms in the multivariable models was not possible. Finally, the validity of the reported models for quantitative EEG analysis should be further tested on an independent new dataset.

To conclude, in a prospectively studied cohort of neonates and young infants with TSC, an abnormal early EEG, and more specifically a dysmature EEG background was associated with a higher probability of ASD traits at the age of 24 months. A dysmature EEG background was also associated with lower DQs on cognitive, language, and motor BSID-III test results. Our findings suggest that detection of early EEG abnormalities in TSC infants can assist in the prediction of neurodevelopmental outcomes, facilitating early diagnosis and intervention.

## Data Availability Statement

The raw data supporting the conclusions of this article will be made available by the authors, without undue reservation.

## Ethics Statement

The studies involving human participants were reviewed and approved by Ethische Commissie UZ/KU Leuven. Written informed consent to participate in this study was provided by the participants' legal guardian/next of kin.

## Author Contributions

JD and LL contributed to study design. JV, KK, RM, PC, BW, KR, MF, PK, RN, AJ, KW, DD-P, MK-F, CH, CF, SS, BB, EA, DK, FJ, and SJ contributed to data collection. JD, ML, BV, JV, KL, SV, and LL contributed to data analysis. JD, ML, SV, and LL contributed to data interpretation and contributed to writing. JD contributed to literature search. All authors contributed to read and approved the submitted version.

## Conflict of Interest

KW was employed by company Transition Technologies. The remaining authors declare that the research was conducted in the absence of any commercial or financial relationships that could be construed as a potential conflict of interest.

## Supplementary Material

Supplementary Material, including more detailed methods for quantitative EEG analysis and Supplementary Tables, are added in a separate file.
