# Supplementary material for: Prediction of Neurodevelopment in Infants With Tuberous Sclerosis Complex Using Early EEG Characteristics
Source: Front Neurol. 2020 Oct 16;11:582891. doi: 10.3389/fneur.2020.582891 (PMC7596378; doi:10.3389/fneur.2020.582891)
Supplement: Supplementary file 1 [file Data_Sheet_1.DOCX]

Supplementary Material

**1 Methods - quantitative EEG analysis**

In order to prove quantitatively that a relation between cerebral dysmaturity patterns and ASD symptoms exists, as observed in clinical practice, we performed a quantitative EEG analysis. This analysis estimates the maturity of the neonatal brain based on EEG analysis and relates it to the ASD outcome.

As reported in Finn et al., the spectral power of the neonatal EEG follows a power-law, i.e. an exponential decay in spectral power, mostly concentrated in the $\delta=\left[ 0\cdot5-4 \right] Hz$ band, which accounts for more than 80% of the power in the EEG.^1^ The exponential decay behaviour is the result of the discontinuity in the neonatal EEG, especially during quiet sleep and has been confirmed by different metrics.^2^ Specifically, this relation between the power-law pattern on the EEG and the discontinuity has been shown by the regularity analysis with Higuchi method, and by using a multifractality framework.^3,4^ Furthermore, a direct association between EEG continuity and irregularity with maturation has been proven by means of multiscale entropy.^5^

In order to assess brain maturation in the population under study and to predict ASD outcome, four groups of features were derived from EEG to describe dysmaturity patterns as defined by Pavlidis et al.:^6^

1. The power in the typical frequency bands of the EEG
2. Quantitative EEG features obtained with the NEURAL toolbox^7^
3. Entropic features to investigate complexity of EEG^5^
4. Fractal features to assess regularity of EEG^4^

The pipeline followed for this analysis was as follows. First, the EEG signals were preprocessed in order to remove artefacts and to improve the quality of the signals. Second, EEG features were extracted in order to quantify maturity of neonatal brain. Finally, the maturity features were related to ASD outcome.

**1.1 EEG preprocessing**

EEG was measured using the 20-10-20 system with reference electrode Cz, after which the EEG was reduced to an 8 channels configuration (Fp1, Fp2, C3, C4, T4, T3, O1, O2) for the quantitative analysis. Both wake and sleep state EEG recordings were analysed. Since the dysmaturity patterns are only related to EEG background free of seizure and artefacts, the signal was band-pass filtered between [1-32] Hz (FIR filter, with limited ripple and 40 db attenuation) and resampled at 64 Hz. Each channel was then processed in non-overlapping windows (apart from some exceptions), which were included if and only if the following criteria were satisfied: standard deviation below 50 $\mu V$, absolute difference sample-to-sample below 50 $\mu V$ and absolute amplitude below 200 $\mu V$.^8^ In case of the NEURAL features, the original preprocessing code has been modified to include the standard deviation criteria. The window size for quantifying the different features were the following:

1. Power analysis: 30 seconds
2. Multiscale entropy: 150 seconds
3. Multifractal features: 150 seconds
4. NEURAL features: 2 seconds for spectral features with 50% overlap, 2 seconds for amplitude features without overlap and 4 seconds for connectivity features with 75% overlap

**1.2 EEG features**

**1.2.1 Power analysis**

The power spectral density (PSD) has been estimated with the non-parametric Welch approach, with 70% overlap and subwindow of 4 seconds. The power has been estimated as integration of the PSD in the following frequency bands $\delta_{1}=\left( 0\cdot5-2 \right] Hz,$ $\delta_{2}=\left( 2-4 \right] Hz$, $\theta=\left( 4-8 \right] Hz, \alpha=\left( 8-16 \right] Hz,$ $\beta=\left( 16-32 \right] Hz$. The power features were log-transformed for each channel and grand-averaged for each recording.

**1.2.2 Entropic features**

The neonatal EEG can be represented as a power-law signal, which has a high-degree of self-similarity.^4,9,10^ This type of signals can be characterized by the so-called Sample Entropy $SampEn$, which measures how predictable the signal is given its past samples. Generally this is done by the calculation of the probability between similar patterns inside the signals. This procedure relies on the counts of m-long templates matching within a certain tolerance $r$(usually defined as 20% the standard deviation of the signal).^11^ $SampEn$ has been used successfully in the prediction of sepsis in premature infants, as well as to evaluate the impact of Kangaroo Care on sleep quality.^12,13^

However, due to the multi-fractal nature of EEG signals, to measure irregularities across different scales, Costa el al. proposed the multiscale sample entropy (MSE).^14^ This methodology can be summarized as the computation of sample entropy across different scales. To obtain the different scales the signals are coarse-graining, i.e at scale$\tau$ the signal is divided in non-overlapping windows with length $\tau$, and each segment is replaced by one value corresponding to the average of that segment.^15^ The MSE is then defined as the $SampEn$ for each coarse-grained signal, and is therefore a function of the scale $\tau.$

The main features derived in this study to describe the dysmaturity patterns where:

1. $SampEn$ for different lengths $m$ of the template
2. The MSE at scale 3 and 20 (i.e. $MSE(\tau=3)$ and $MSE(\tau=20)$)
3. The complexity index $CI= \sum_{\tau} MSE(\tau)$, which is the area under the MSE curve

The SampEn and $MSE(\tau=3)$ represents the information at small scales or high frequency, while $MSE(\tau=20)$ represents information at longer scales or lower frequency. $CI$ is a general measure of irregularities across scales. All features were estimated in nonoverlapping windows and grand averaged for each recording.

**1.2.3 Fractality features**

Signals with a high degree of self-similarity are also known as fractals or scale-free signals. Those type of signals are characterized by long-exponential decay autocorrelation function (ACF) as well as a power-law spectrum. Both ACF and the spectrum are controlled by the Hurst exponent (H), which defines the rate of decay and therefore measures the level of similarity.^16^ An efficient way to estimate H is based on the wavelet transform.^16^ However, complex and discontinuous signals can vary in fractal properties over time, i.e. the Hurst exponent and therefore the rate of ACF decay can differ.^17^ Therefore, Wendt et al. proposed to estimate the spectrum of singularities *(SS),* which measures the different Hurst Exponents in the signal and the associated fractal dimension.^18^ Specifically, the authors defined a set of parameters to describe SS:

1. The main Hurst exponent H as the location of the SS maximum, which represents the main regularity in the signal
2. The parameters $C_{2}$ and $C_{3}$, which respectively represent the width and the asymmetry of SS, measuring implicitly the “amount of fractals” inside the time series.
3. The difference between maximal and minimal Hurst exponents $\Delta H$ of the SS, which resembles $C_{2}$ and assesses the number of singularities in a signal. The higher the number of singularities, the higher the discontinuity.

These features were computed by means of the WLBFM toolbox.^18^ All features were estimated in non-overlapping windows and grand averaged for each recording.

**1.2.4 NEURAL features**

Quantification of dysmature EEG features has already been investigated by Toole et al., who proposed the NEURAL toolbox available on GitHub.^7^ The approach proposed by the authors yields an exhaustive range of features that can be obtained from amplitude, Burst and spectral information, connectivity analysis and the range EEG (rEEG). The latter represents one of the standardized estimation of the amplitude integrated EEG (aEEG), since there is no clear definition of aEEG and there exist multiple versions of the algorithm to compute it.^7^  Among all the attributes, it is worth to mention that rEEG was used to quantify the level of dysmaturity, by means of its  lower and upper margin and the rEEG asymmetry. These two margins represent respectively the 5^th^ and 95^th^ percentiles of the rEEG, while the rEEG asymmetry expresses the difference in distance from the median to these two margins. The rEEG can be estimated both for the full-band signal as well as for the common individual frequency bands in the EEG. More details are reported in Toole et al.^7^ As the previous features, the obtained features were then grand-average for each recording.

**1.3 ASD symptoms prediction**

We selected the five most discriminant features to classify ASD outcome. We select five features in order to guarantee that the number of predictors is below 10% of the total number of patients.^19^ The total number of patients with ASD assessment at 24 months was 63, but two of them could not be preprocessed. Hence, the number of patients was reduced to 61.

For the prediction of ASD symptoms, a binary classification model was developed using the five features with highest ratio between higher inter-group variance and lower intra-group variance.^20^ We built several models for the classifier, including support vector machines (SVM), k nearest neighbour (kNN), and a Linear discriminant analysis (LDA). The LDA classifier was tested with 3-fold testing (2/3 of data as training set and 1/3 test set) and the hyperparameters were tuned using 10-fold cross-validation (9/10 of the training set is used for training set and 1/10 is used as validation set). The classification results were reported as classification error and area under the curve (AUC, intended as measure of accuracy). The classifier showing the best performance-versus-complexity ratio was LDA: the results of this classifier are presented in the main text.

**1.4 References**

1. Finn D, O'Toole JM, Dempsey EM, Boylan GB. EEG for the assessment of neurological function in newborn infants immediately after birth. Arch Dis Child Fetal Neonatal Ed. 2019;104(5):F510-F4. doi: 10.1136/archdischild-2018-315231.

2. Andre M, Lamblin MD, d'Allest AM, Curzi-Dascalova L, Moussalli-Salefranque F, T SNT, et al. Electroencephalography in premature and full-term infants. Developmental features and glossary. Neurophysiol Clin. 2010;40(2):59-124. doi: 10.1016/j.neucli.2010.02.002.

3. O'Toole JM, Pavlidis E, Korotchikova I, Boylan GB, Stevenson NJ. Temporal evolution of quantitative EEG within 3 days of birth in early preterm infants. Sci Rep. 2019;9(1):4859. doi: 10.1038/s41598-019-41227-9.

4. Lavanga M, De Wel O, Caicedo A, Heremans E, Jansen K, Dereymaeker A, et al. Automatic quiet sleep detection based on multifractality in preterm neonates: Effects of maturation. Conf Proc IEEE Eng Med Biol Soc. 2017;2017:2010-3. doi: 10.1109/embc.2017.8037246.

5. De Wel O, Lavanga M, Caicedo A, Jansen K, Dereymaeker A, Naulaers G, et al. Complexity Analysis of Neonatal EEG Using Multiscale Entropy: Applications in Brain Maturation and Sleep Stage Classification. Entropy. 2017;19:516. doi: 10.3390/e19100516.

6. Pavlidis E, Lloyd RO, Boylan GB. EEG - A Valuable Biomarker of Brain Injury in Preterm Infants. Dev Neurosci. 2017;39(1-4):23-35. doi: 10.1159/000456659.

7. O' Toole J, Boylan G. NEURAL: quantitative features for newborn EEG using Matlab [updated 20 April 2017; cited 2017 20 April 2017]. Available from: <https://arxiv.org/pdf/1704.05694.pdf>.

8. Isler JR, Stark RI, Grieve PG, Welch MG, Myers MM. Integrated information in the EEG of preterm infants increases with family nurture intervention, age, and conscious state. PLoS One. 2018;13(10):e0206237. doi: 10.1371/journal.pone.0206237.

9. Kesic S, Spasic SZ. Application of Higuchi's fractal dimension from basic to clinical neurophysiology: A review. Comput Methods Programs Biomed. 2016;133:55-70. doi: 10.1016/j.cmpb.2016.05.014.

10. Popivanov D, Stomonyakov V, Minchev Z, Jivkova S, Dojnov P, Jivkov S, et al. Multifractality of decomposed EEG during imaginary and real visual-motor tracking. Biol Cybern. 2006;94(2):149-56. doi: 10.1007/s00422-005-0037-5.

11. Lake DE, Moorman JR. Accurate estimation of entropy in very short physiological time series: the problem of atrial fibrillation detection in implanted ventricular devices. Am J Physiol Heart Circ Physiol. 2011;300(1):H319-25. doi: 10.1152/ajpheart.00561.2010.

12. Lake DE, Richman JS, Griffin MP, Moorman JR. Sample entropy analysis of neonatal heart rate variability. Am J Physiol Regul Integr Comp Physiol. 2002;283(3):R789-97. doi: 10.1152/ajpregu.00069.2002.

13. Kaffashi F, Scher MS, Ludington-Hoe SM, Loparo KA. An analysis of the kangaroo care intervention using neonatal EEG complexity: a preliminary study. Clin Neurophysiol. 2013;124(2):238-46. doi: 10.1016/j.clinph.2012.06.021.

14. Costa M, Goldberger AL, Peng CK. Multiscale entropy analysis of complex physiologic time series. Phys Rev Lett. 2002;89(6):068102. doi: 10.1103/PhysRevLett.89.068102.

15. Hu M, Liang H. Multiscale Entropy: Recent Advances. Complexity and Nonlinearity in Cardiovascular Signals: Springer; 2017. p. 115-38.

16. Doret M, Spilka J, Chudacek V, Goncalves P, Abry P. Fractal Analysis and Hurst Parameter for Intrapartum Fetal Heart Rate Variability Analysis: A Versatile Alternative to Frequency Bands and LF/HF Ratio. PLoS One. 2015;10(8):e0136661. doi: 10.1371/journal.pone.0136661.

17. Jaffard S, Lashermes B, Abry P, editors. Wavelet Leaders in Multifractal Analysis2007; Basel: Birkhäuser Basel.

18. Wendt H, Abry P, Jaffard S. Bootstrap for Empirical Multifractal Analysis. IEEE Signal Processing Magazine. 2007;24(4):38-48. doi: 10.1109/MSP.2007.4286563.

19. Floyd S, Warmuth M. Sample Compression, Learnability, and the Vapnik-Chervonenkis Dimension. Machine Learning. 1995;21(3):269-304. doi: 10.1023/A:1022660318680.

20. Chen Y-W, Lin C-J. Combining SVMs with Various Feature Selection Strategies. In: Guyon I, Nikravesh M, Gunn S, Zadeh LA, editors. Feature Extraction: Foundations and Applications. Berlin, Heidelberg: Springer Berlin Heidelberg; 2006. p. 315-24.

**Supplementary table 1**

|  | **No ASD risk**  **(N = 44)** | **ASD risk**  **(N = 19)** | **p-value** | **OR**  **(unadjusted)** | **95% CI** |
| --- | --- | --- | --- | --- | --- |
| **Non-EEG characteristics** |  | | | | |
| GA at birth  Median (IQR) in weeks | 38 ^0/7^  [37 ^1/7^ – 39 ^6/7^] | 38 ^3/7^  [37 ^0/7^ – 40 ^0/7^] | 0.517 |  |  |
| Sex |  | | | | |
| Male  (N = 35) | 22  (63%) | 13  (37%) | 0.181 |  |  |
| Female  (N = 28) | 22  (79%) | 6  (21%) |  | 0.4 | 0.1 – 1.4 |
| Mutation |  | | | | |
| Mutation in *TSC2*  (N = 45) | 31  (69%) | 14  (31%) | 0.897 | 1.1 | 0.3 – 3.7 |
| Mutation in *TSC1*  (N = 17) | 12  (71%) | 5  (29%) |  |  |  |
| Treatment |  | | | | |
| Preventive treatment  (N = 19) | 13  (68%) | 6  (32%) | 0.872 |  |  |
| Conventional treatment  (N = 44) | 31  (70%) | 13  (30%) |  | 0.9 | 0.3 – 2.9 |
| GA at first EEG  Median (IQR) in weeks | 43 ^0/7^  [40 ^5/7^ – 46 ^4/7^] | 41 ^5/7^  [39 ^0/7^ – 43 ^5/7^] | 0.144 |  |  |
| **Other outcome measures** |  | | | | |
| DQ cognitive BSID-III  Median [IQR] | 82.5  [70 – 95.75] | 65  [55 – 80] | 0.006 |  |  |
| DQ language BSID-III  Median [IQR] | 71  [65 – 83] | 62  [50 – 68] | <0.001 |  |  |
| DQ motor BSID-III  Median [IQR] | 79  [70 – 92]) | 67  [55 – 73] | 0.001 |  |  |
| **EEG biomarkers** |  | | | | |
| **Normal vs abnormal EEG** |  | | | | |
| Normal EEG  (N = 27) | 23  (85%) | 4  (15%) | 0.027 |  |  |
| Abnormal EEG  (N = 36) | 21  (58%) | 15  (42%) |  | 4.1 | 1.2-14.4 |
| **IED** |  | | | | |
| Absence of IED  (N=35) | 27  (77%) | 8  (23%) | 0.162 |  |  |
| Presence of IED  (N=28) | 17  (61%) | 11  (39%) |  | 2.2 | 0.7 –6.5 |
| **Background** |  | | | | |
| Normal EEG background  (N =41) | 32  (78%) | 9  (22%) | 0.057 |  |  |
| Abnormal EEG background  (N = 22) | 12  (55%) | 10  (45%) |  | 3.0 | 0.97 – 9.1 |
| No focal slowing  (N = 49) | 35  (71%) | 14  (29%) | 0.608 |  |  |
| Focal slowing  (N = 14) | 9  (64%) | 5  (36%) |  | 1.4 | 0.4 – 4.8 |
| Mature EEG background  (N =49) | 38  (78%) | 11  (22%) | 0.017 |  |  |
| Dysmature EEG background  (N = 14) | 6  (43%) | 8  (57%) |  | 4.6 | 1.3 – 16.1 |

Supplementary table 1: Description of non-EEG characteristics and EEG biomarkers between infants without and with autism spectrum disorder (ASD) traits at the age of 24 months. Continuous non-EEG characteristics (GA at birth, GA at first EEG and DQs) were assessed by Mann-Whitney U test. Binary non-EEG characteristics and EEG biomarkers were assessed using univariable logistic regression analysis. GA: gestational age, IQR: interquartile range, DQ: developmental quotient, BSID-III: Bayley Scales of Infant and Toddler Development III, IED: interictal epileptiform discharge, OR: odds ratio, 95%CI: 95% confidence interval.

**Supplementary table 2**

|  | **Median cognitive DQ**  **at 24 months** | **IQR** | **F-value** | **p-value** |
| --- | --- | --- | --- | --- |
| **Non-EEG characteristics** |  | | | |
| Sex |  | | | |
| Male  (N = 35) | 75.00 | 65.00 – 91.00 | NA | 0.353 |
| Female  (N = 29) | 70.00 | 55.00 – 92.50 |  |  |
| Mutation |  | | | |
| Mutation in *TSC2*  (N = 46) | 70.00 | 58.75 – 85.00 | NA | 0.006 |
| Mutation in *TSC1*  (N = 17) | 90.00 | 72.50 – 100.00 |  |  |
| Treatment |  | | | |
| Preventive treatment  (N = 19) | 75.00 | 65.00 – 95.00 | NA | 0.836 |
| Conventional treatment  (N = 45) | 75.00 | 62.50 – 90.50 |  |  |
| **EEG characteristics** |  | | | |
| **Normal vs abnormal EEG** |  | | | |
| Normal EEG  (N = 27) | 80.00 | 65.00 – 100.00 | 2.387 | 0.127 |
| Abnormal EEG  (N = 37) | 70.00 | 60.00 – 90.00 |  |  |
| **IED** |  | | | |
| Absence of IED  (N=36) | 77.50 | 65.00 – 95.75 | 1.464 | 0.231 |
| Presence of IED  (N=28) | 70.00 | 61.25 – 90.00 |  |  |
| **Background** |  | | | |
| Normal EEG background  (N=41) | 80.00 | 67.50 – 95.00 | 4.998 | 0.029 |
| Abnormal EEG background  (N=23) | 70.00 | 55.00 – 80.00 |  |  |
| No focal slowing  (N=49) | 75.00 | 65.00 – 93.00 | 1.088 | 0.301 |
| Focal slowing  (N=15) | 70.00 | 60.00 – 90.00 |  |  |
| Mature EEG background  (N=50) | 75.00 | 65.00 – 95.00 | 4.981 | 0.029 |
| Dysmature EEG background  (N=14) | 62.50 | 55.00 – 80.00 |  |  |

Supplementary table 2: Cognitive DQ based on the BSID-III results at the age of 24 months. The relation between non-EEG characteristics and the DQ was assessed by Mann-Whitney U test. The relation between EEG biomarkers and the cognitive DQ was studied using univariable linear models. Since the cognitive DQs were not normally distributed, the DQs were logarithmic transformed. IQR: interquartile range, DQ: developmental quotient, BSID-III: Bayley Scales of Infant and Toddler Development III, IED: interictal epileptiform discharge, NA: not applicable.

**Supplementary table 3**

|  | **F-value** | **p-value** |
| --- | --- | --- |
| **Part A** | | |
| Abnormal vs normal EEG | 0.658 | 0.420 |
| Conventional vs preventive treatment | 0.737 | 0.394 |
| *TSC2* vs *TSC1* mutation | 8.919 | 0.004 |
| **Part B** | | |
| Abnormal vs normal EEG | 0.200 | 0.657 |
| Abnormal vs normal EEG background | 2.999 | 0.089 |
| Conventional vs preventive treatment | 0.888 | 0.350 |
| *TSC2* vs *TSC1* mutation | 9.362 | 0.003 |
| **Part C** |  |  |
| Abnormal vs normal EEG | 0.177 | 0.676 |
| Abnormal vs normal EEG background | 1.197 | 0.278 |
| Dysmature vs mature EEG background | 0.150 | 0.700 |
| Conventional vs preventive treatment | 0.662 | 0.419 |
| *TSC2* vs *TSC1* mutation | 8.447 | 0.005 |

Supplementary table 3: The relation between EEG biomarkers and the cognitive DQ based on the BSID-III results at 24 months were assessed using multivariable linear models. Since the cognitive DQs were not normally distributed, the DQs were logarithmic transformed. Part A: multivariable model including abnormal vs normal EEG, the treatment strategy and the mutation as predictor variables. Part B: multivariable model including background abnormalities, treatment strategy and mutation as predictor variables. Part C: multivariable model including the maturation of the EEG background, treatment strategy and mutation as predictor variables. DQ: developmental quotient, BSID-III: Bayley Scales of Infant and Toddler Development III, IED: interictal epileptiform discharge.

**Supplementary table 4**

|  | **Language DQ at 24 months** | | | | **Motor DQ at 24 months** | | | |
| --- | --- | --- | --- | --- | --- | --- | --- | --- |
|  | **Median DQ** | **IQR** | **F-value** | **p-value** | **Median DQ** | **IQR** | **F-value** | **p-value** |
| **Non-EEG characteristics** |  | | | |  | | | |
| Sex |  | | | |  | | | |
| Male  (N = 35) | 68.00 | 62.00 – 77.00 | NA | 0.739 | 73.00 | 67.00 – 85.00 | NA | 0.708 |
| Female  (N = 28) | 66.50 | 56.75 – 77.00 |  |  | 73.00 | 58.75 – 84.25 |  |  |
| Mutation |  | | | |  | | | |
| Mutation in *TSC2*  (N = 45) | 65.00 | 59.00 – 71.00 | NA | 0.053 | 70.00 | 59.50 - 79.00 | NA | 0.002 |
| Mutation in *TSC1*  (N = 17) | 74.00 | 62.00 – 95.50 |  |  | 82.00 | 73.00 – 98.50 |  |  |
| Treatment |  | | | |  | | | |
| Preventive treatment  (N = 18) | 66.50 | 61.25 – 73.00 | NA | 0.784 | 77.50 | 67.00 – 82.75 | NA | 0.578 |
| Conventional treatment  (N = 45) | 68.00 | 59.00 - 77.50 |  |  | 73.00 | 62.00 – 87.00 |  |  |
| **EEG biomarkers** |  | | | |  | | | |
| **Normal vs abnormal EEG** |  | | | |  | | | |
| Normal EEG  (N = 26) | 69.50 | 62.00 – 95.00 | 5.702 | 0.020 | 76.00 | 69.75 - 82.00 | 2.552 | 0.115 |
| Abnormal EEG  (N = 37) | 65.00 | 56.00 – 75.00 |  |  | 70.00 | 59.50 – 82.00 |  |  |
| **IED** |  | | | |  | | | |
| Absence of IED  (N=35) | 68.00 | 62.00 – 94.00 | 3.750 | 0.057 | 73.00 | 67.00 – 85.00 | 1.559 | 0.217 |
| Presence of IED  (N=28) | 65.00 | 56.00 – 75.50 |  |  | 70.00 | 58.00 – 84.25 |  |  |
| **Background** |  | | | |  | | | |
| Normal EEG background  (N=40) | 71.00 | 62.00 – 81.75 | 7.989 | 0.006 | 76.00 | 67.50 – 89.00 | 4.298 | 0.042 |
| Abnormal EEG background  (N=23) | 59.00 | 53.00 -71.00 |  |  | 70.00 | 55.00 – 79.00 |  |  |
| No focal slowing  (N=48) | 68.00 | 62.00 – 77.75 | 1.231 | 0.272 | 73.00 | 67.00 – 85.00 | 0.308 | 0.581 |
| Focal slowing  (N=15) | 65.00 | 56.00 – 74.00 |  |  | 70.00 | 58.00 – 82.00 |  |  |
| Mature EEG background  (N=49) | 68.00 | 62.00 – 79.00 | 11.234 | 0.001 | 76.00 | 67.00 - 87.00 | 5.993 | 0.017 |
| Dysmature EEG background  (N=14) | 59.00 | 49.50 – 68.75 |  |  | 67.00 | 48.25 – 74.50 |  |  |

Supplementary table 4: Language and motor DQ based on the BSID-III results 24 months. The relation between non-EEG characteristics and the DQs was assessed by Mann-Whitney U test. The relation between EEG biomarkers and the language and motor DQs were analysed using univariable linear models. Since the language DQs were not normally distributed, the DQs were reciprocal transformed. IQR: interquartile range, DQ: developmental quotient, BSID-III: Bayley Scales of Infant and Toddler Development III, IED: interictal epileptiform discharge, NA: not applicable.

**Supplementary table 5**

|  | **F-value** | **p-value** |
| --- | --- | --- |
| **Part A** | | |
| Abnormal vs normal EEG | 3.306 | 0.074 |
| Conventional vs preventive treatment | 0.006 | 0.940 |
| *TSC2* vs *TSC1* mutation | 3.673 | 0.060 |
| **Part B** | | |
| Abnormal vs normal EEG | 0.140 | 0.710 |
| Abnormal vs normal EEG background | 2.928 | 0.093 |
| Conventional vs preventive treatment | 0.022 | 0.883 |
| *TSC2* vs *TSC1* mutation | 3.883 | 0.054 |
| **Part C** |  |  |
| Abnormal vs normal EEG | 0.222 | 0.639 |
| Abnormal vs normal EEG background | 0.101 | 0.752 |
| Dysmature vs mature EEG background | 2.706 | 0.106 |
| Conventional vs preventive treatment | 0.065 | 0.800 |
| *TSC2* vs *TSC1* mutation | 2.722 | 0.105 |

Supplementary table 5: The relation between EEG biomarkers and the language DQ based on the BSID-III results at 24 months was assessed using multivariable linear models. Since the language DQs were not normally distributed, the DQs were reciprocal transformed. Part A: multivariable model including abnormal vs normal EEG, the treatment strategy and the mutation as predictor variables. Part B: multivariable model including background abnormalities, treatment strategy and mutation as predictor variables. Part C: multivariable model including the maturation of the EEG background, treatment strategy and mutation as predictor variables. DQ: developmental quotient, BSID-III: Bayley Scales of Infant and Toddler Development III, IED: interictal epileptiform discharge.

**Supplementary table 6**

|  | **F-value** | **p-value** |
| --- | --- | --- |
| **Part A** | | |
| Abnormal vs normal EEG | 0.666 | 0.418 |
| Conventional vs preventive treatment | 1.121 | 0.294 |
| *TSC2* vs *TSC1* mutation | 11.723 | 0.001 |
| **Part B** | | |
| Abnormal vs normal EEG | 0.088 | 0.767 |
| Abnormal vs normal EEG background | 2.270 | 0.137 |
| Conventional vs preventive treatment | 1.279 | 0.263 |
| *TSC2* vs *TSC1* mutation | 12.115 | 0.001 |
| **Part C** |  |  |
| Abnormal vs normal EEG | 0.064 | 0.802 |
| Abnormal vs normal EEG background | 0.462 | 0.500 |
| Dysmature vs mature EEG background | 0.605 | 0.440 |
| Conventional vs preventive treatment | 0.818 | 0.370 |
| *TSC2* vs *TSC1* mutation | 10.609 | 0.002 |

Supplementary table 6: The relation between EEG biomarkers and motor DQ based on the BSID-III results at 24 months was assessed using multivariable linear models. Part A: multivariable model including abnormal vs normal EEG, the treatment strategy and the mutation as predictor variables. Part B: multivariable model including background abnormalities, treatment strategy and mutation as predictor variables. Part C: multivariable model including the maturation of the EEG background, treatment strategy and mutation as predictor variables. DQ: developmental quotient, BSID-III: Bayley Scales of Infant and Toddler Development III, IED: interictal epileptiform discharge.
